# P-590. Go with the Flow: An Early Look at a Pilot High Throughput Metagenomic Wastewater Analysis for Antimicrobial Resistance in a Large Military Treatment Facility

**DOI:** 10.1093/ofid/ofaf695.804

**Published:** 2026-01-11

**Authors:** Paige Salerno, Wesley Campbell, Valerie J Morley, Nora Watson, Marleen M Welsh, Michael Backlund, Tyler Moeller, Michael Zamani, Casandra Philipson, Dawn Gratalo, Beth Higa Roberts, Tisza A S Bell, Diego Insausti, Benjamin Knisely, Melissa Austin, Paige E Waterman

**Affiliations:** Ginkgo Bioworks, Andover, MA; Walter Reed National Military Medical Center, Bethesda, Maryland; Ginkgo Biosecurity, Albuquerque, NewMexico; Walter Reed National Military Medical Center, Bethesda, Maryland; Booz Allen Hamilton, Bethesda, Maryland; WRNMMC, Bethesda, Maryland; Naval Medical Research Unit 6, Maryland, Maryland; Walter Reed National Military Medical Center, Bethesda, Maryland; Ginkgo Bioworks, Andover, MA; Ginkgo Bioworks, Andover, MA; Ginkgo Biosecurity, Albuquerque, NewMexico; Booz Allen Hamilton, Bethesda, Maryland; BOOZ ALLEN HAMILTON, MIAMI SPRINGS, Florida; Booz Allen Hamilton, Bethesda, Maryland; Walter Reed National Military Medical Center, Bethesda, Maryland; USUHS, Bethesda, Maryland

## Abstract

**Background:**

Wastewater surveillance (WWS) is increasingly used as a complementary public health tool to case reporting; however, its use outside of identifying viral pathogens and its ability to link to clinical findings is limited. Presented here are early antimicrobial resistant (AMR) WWS findings focused on key drug-resistant pathogens at a large Military Treatment Facility (MTF) correlated with clinical antimicrobial susceptibility (AST) results.

**Figure:**
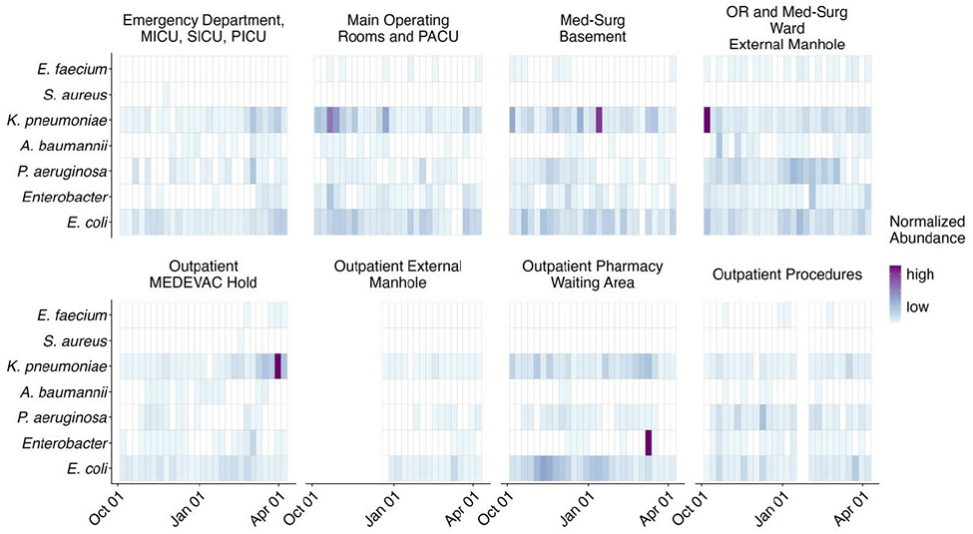
Building Level ESKAPEE Detection Displayed as Normalized Abundance RPKM ESKAPEE: E. faecium, S. aureus, K. pneumoniae, A. baumannii, P. aeruginosa, Enterobacter spp, and E. coli; RPKM: Reads Per Kilobase Per Million Mapped Reads; MICU: Medical Intensive Care Unit; SICU: Surgical Intensive Care Unit; PICU: Pediatric Intensive Care Unit; Med-Surg: Medical Surgical Ward Building; PACU: Post Anesthesia Care Unit

**Figure:**
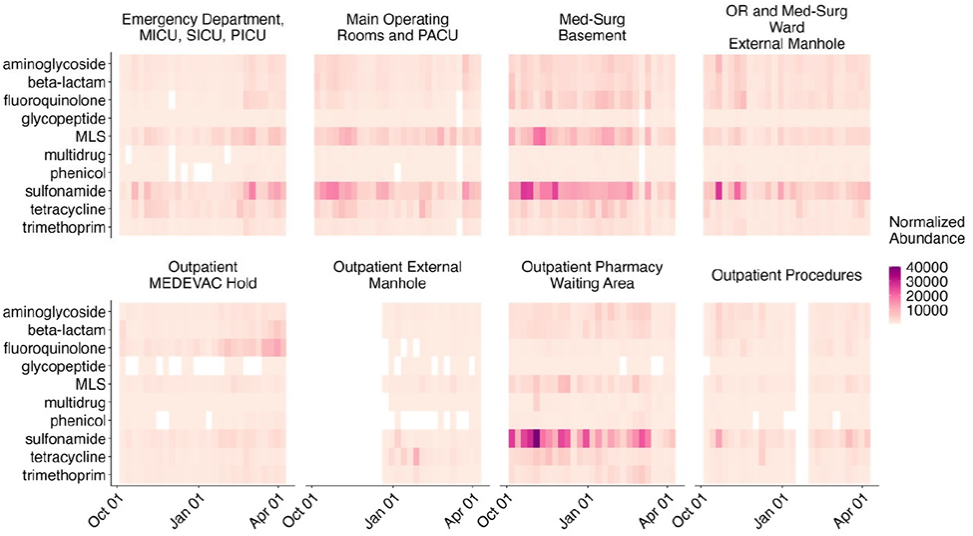
Building Level Antimicrobial Resistance Gene Detection as Normalized Abundance RPKM RPKM: Reads Per Kilobase Per Million Mapped Reads; MICU: Medical Intensive Care Unit; SICU: Surgical Intensive Care Unit; PICU: Pediatric Intensive Care Unit; Med-Surg: Medical Surgical Ward or Building

**Methods:**

WWS at Walter Reed National Military Medical Center (WRNMMC) was conducted 3-times weekly from October 2024 through April 2025 at 8 outpatient, inpatient, and MEDEVAC housing effluent access points. Samples were concentrated, then nucleic acid was extracted for analysis of over 180 bacterial pathogens and 1200+ markers of AMR using specialized bioinformatics programming. Clinical AST for *E. faecium*, *S. aureus*, *K. pneumoniae*, *A. baumannii*, *P. aeruginosa*, *Enterobacter* spp, and *E. coli.* (ESKAPEE) and patient demographics and specimen characteristics were extracted for WRNMMC MTF patients.

**Results:**

AMR-WWS findings are reported with frequency counts of detection along with genetic abundance showing significant correlation of detection with inpatient wards (Figure 1). 98% of 540 samples tested positive for at least one ESKAPEE pathogen, 100% contained at least one AMR gene (Figure 2). Clinical AST findings of 1,713 samples include 688 (40.2%) as inpatient collected, with 51 (6.0%) of 847 ESKAPEE isolates identified as multi-drug resistant. These clinical AST AMR results correlate to inpatient nursing units undergoing WWS genomic testing. Outpatient clinic and MEDEVAC hold effluent represents the lowest detection AMR markers. Further assessment of AMR definitions and analysis of detection of key pathogens associated with clinical care is ongoing.

**Conclusion:**

AMR-WWS with linkage to clinical AST provides a strong foundation to establish baseline pathogen presence, resistance, and building of an early warning system for potential case clusters or significant public health events. Analysis is ongoing to support predictive modeling and advanced analytics.

**Disclosures:**

Valerie J. Morley, PhD, Ginkgo Bioworks: employee|Ginkgo Bioworks: Stocks/Bonds (Public Company) Marleen M. Welsh, Ph.D., Altria Group Inc: Stocks/Bonds (Public Company)|Johnson & Johnson: Stocks/Bonds (Public Company)|Merk & Co Inc: Stocks/Bonds (Public Company)|Pfizer Inc: Stocks/Bonds (Public Company)|Solventum Corp: Stocks/Bonds (Public Company) Casandra Philipson, PhD, PhD, Ginkgo Bioworks: Stocks/Bonds (Public Company) Dawn Gratalo, MS, Ginkgo Bioworks: Stocks/Bonds (Public Company)

